# Dysbiosis of Gut Microbiota Promotes Hepatocellular Carcinoma Progression by Regulating the Immune Response

**DOI:** 10.1155/2021/4973589

**Published:** 2021-10-20

**Authors:** Nan Zhang, Yusong Gou, Shan Liang, Ning Chen, Yali Liu, Qiushui He, Jing Zhang

**Affiliations:** ^1^Department of Medical Microbiology, Capital Medical University, No. 10, Youwai Xitoutiao Street, Fengtai District, Beijing 100069, China; ^2^The Third Unit, Department of Hepatology, Beijing Youan Hospital, Capital Medical University, No. 8 Youwai Xitoutiao Street, Fengtai District, Beijing 100069, China; ^3^Department of Medical Microbiology, University of Turku, Kiinamyllynkatu 10, Turku 20520, Finland

## Abstract

**Method:**

This study included 74 Chinese male patients with HCC. They were divided into early (*n* = 19), intermediate (*n* = 37), and terminal (*n* = 18) groups, referred to as Barcelona Clinic Liver Cancer stage 0+A, B, and C+D, respectively. Paired fecal and plasma samples were collected. Microbial composition and profiles were analyzed by 16S rRNA gene sequencing. The levels of gut damage marker (regenerating islet-derived protein 3*α* (REG3*α*)) and microbial translocation markers (soluble CD14 (sCD14), lipopolysaccharide-binding protein (LBP), peptidoglycan recognition proteins (PGRPs)) were determined in plasma samples of patients by ELISA. Twenty plasma cytokine and chemokines were determined by Luminex.

**Results:**

In early, intermediate, and terminal groups, the abundance of the *Bifidobacteriaceae* family decreased significantly (3.52%, 1.55%, and 0.56%, respectively, *P* = 0.003), while the abundance of the *Enterococcaceae* family increased significantly (1.6%, 2.9%, and 13.4%, respectively, *P* = 0.022). Levels of REG3*α* and sCD14 were markedly elevated only in the terminal group compared with the early (*P* = 0.025 and *P* = 0.048) and intermediate groups (*P* = 0.023 and *P* = 0.046). The level of LBP significantly increased in the intermediate (*P* = 0.035) and terminal (*P* = 0.025) groups compared with the early group. The PGRP levels were elevated only in the terminal group compared with the early group (*P* = 0.018). The ratio of *Enterococcaceae* to *Bifidobacteriaceae* was significantly associated with the levels of REG3*α*, LBP, sCD14, and PGRPs. With HCC progression, increased levels of inflammatory cytokines accompanied by a T cell-immunosuppressive response and microbial translocation were observed.

**Conclusion:**

Gut microbiota compositional and functional shift, together with elevated gut damage and microbial translocation, may promote HCC development by stimulating inflammatory response and suppressing T cell response.

## 1. Introduction

Hepatocellular carcinoma (HCC) is the third leading cause of cancer mortality worldwide [1]. However, the underlying mechanism is poorly understood. Recently, increasing evidence has indicated the relationship between dysbiosis of gut microbiome and HCC development [2–4]. However, there has been only one report in which the abundance of proinflammatory bacteria *Enterobacteriaceae* tended to increase parallelly with the progression of HCC [5]. The findings demonstrated that microbiota dysbiosis could regulate the innate immune system and advance liver disease [6–8].

Besides microbiome dysbiosis, gut permeability was also found to correlate with HCC. A recent study reported that gut permeability was significantly higher in patients with HCC compared with healthy individuals [9]. The effect of microbial translocation on cirrhosis was well studied [10], but was not researched in HCC. Therefore, this cross-sectional study is aimed at evaluating microbiome dysbiosis, gut damage, and microbial translocation in 74 male patients in different stages of HCC. The levels of cytokines, which reflect inflammation and immune response, were also measured. It was speculated that dysbiosis together with increased microbial translocation might impact HCC progression by regulating systemic inflammation and immune response.

## 2. Materials and Methods

### 2.1. Patient Selection

Patients with HCC admitted to Beijing Youan Hospital from June 2018 to September 2019 were recruited in this study. The study was approved by the ethics committee of Beijing Youan Hospital, Capital Medical University (No. 2018-038). All patients provided written informed consents.

The diagnosis of HCC was made following the international guidelines [11]. The inclusion criteria were as follows: HCC; age ≥ 18 years; male sex; and hepatitis B virus (HBV) surface antigen-positive or anti-hepatitis C virus (anti-HCV) antibody positive. The exclusion criteria were as follows: patients suffering from other causes of liver diseases, such as nonalcoholic fatty liver disease, alcoholic liver disease, and cholestatic liver disease. Patients with irritable bowel syndrome or inflammatory bowel disease; other cancers besides HCC; and autoimmune disease or serious cardiac, kidney, and respiratory diseases were also excluded. No antibiotic, probiotic, and immunosuppressive drugs were taken within 2 weeks before sample collection.

HCC was staged according to the Barcelona Clinic Liver Cancer (BCLC) staging system [12]. Stage 0 referred to single nodule < 2 cm and Child-Pugh A. Stage A was defined as single or three nodules < 3 cm and Child-Pugh A or B. Stage B referred to patients with multinodules and Child-Pugh A or B. Stage C comprised patients with portal invasion or extrahepatic spread and Child-Pugh A or B. Stage D included patients with HCC and Child-Pugh C. In the present study, patients with HCC were divided into early, intermediate, and terminal groups, which referred to stage 0+A, B, and C+D, respectively.

### 2.2. Fecal Sample Collection, DNA Extraction, and Polymerase Chain Reaction Sequencing

Each fresh fecal sample was split into two tubes containing bacterial RNA LOCKER (Youkang, Nanjing, China) and stored at -80°C. Total bacterial DNA was isolated from stool samples using the QIAamp Fast DNA Stool Mini Kit (Qiagen, Hilden, Germany) following the manufacturer's instructions and diluted to 1 ng/*μ*L using sterile water. The V4-V5 regions of the prokaryotic 16S rRNA gene were amplified using the universal primer pair 515F (5′-GTGYCAGCMGCCGCGGTA-3′) and 909R (5′-CCCCGYCAATTCMTTTRAGT-3′) with barcode and then sequenced and analyzed [13]. The reason to choose the V4-V5 regions was that we also wanted to detect low abundant bacteria in the samples [5, 14]. Paired stool samples from 20 patients were sequenced to validate the results. Sequencing libraries were generated using TruSeq DNA Polymerase Chain Reaction- (PCR-) Free Sample Preparation Kit (Illumina, USA), and index codes were added. The amplification products were sequenced on an Illumina HiSeq 2500 platform, and 250 bp paired-end reads were generated. Sequencing data were analyzed using Quantitative Insights into Microbial Ecology (QIIME) platform version 1.9 and R v3.3.1 [15]. The paired-end reads from the DNA fragments were merged using FLASH (v1.2.7) [16].

### 2.3. Operational Taxonomy Unit Clustering and Taxonomy Annotation

Sequences with more than 97% similarity were assigned to the same operational taxonomy units (OTUs). OTU classification was conducted by running a Basic Local Alignment Search Tool (BLAST) search against the Greengenes database using the representative sequence set as a query. A representative sequence for each OTU was screened for further annotation. The sequences were annotated using RDP classifier V.2.6 according to the developer's documents (http://rdp.cme.msu.edu/classifier/class_help.jsp#conf) [17].

### 2.4. Bacterial Diversity Analysis

Bacterial diversity was determined by sampling-based OTU analysis. It is presented by Observed species index, Chao1 index, Shannon index, and Simpson index, all of which were calculated using the R vegan program package [18].

### 2.5. Structure of Microbial Communities and OTU Biomarker Identification

Principal component analysis (PCA) was conducted by R package (http://www.R-project.org/). The weighted and unweighted UniFrac distances were calculated with the phyloseq package [19]. The linear discriminant analysis (LDA) effect size (LEfSe) model was used to identify differences in microbiota composition for phylotypes [20]. Based on the normalized relative abundance matrix, taxa with significantly different abundances were determined by LEfSe using Kruskal-Wallis rank sum test.

### 2.6. Determination of Plasma Biomarkers of Gut Damage and Microbial Translocation

The plasma marker of gut damage, regenerating islet-derived protein (REG3*α*), was determined using the ELISA kit (R&D Systems, ON, Canada). Soluble CD14 (sCD14), lipopolysaccharide-binding protein (LBP), and peptidoglycan recognition proteins (PGRPs) were selected as microbial translocation markers. The ELISA kits were Human CD14 Quantikine ELISA kit (Bio-Techne Ltd., Abingdon, United Kingdom), Human LBP DuoSet ELISA kit (Bio-Techne Ltd.), and Human PGRPs ELISA kit (Thermo Fisher, Merelbeke, Belgium). All assays were performed in duplicate following the manufacturer's protocol.

### 2.7. Determination of Plasma Cytokines and Chemokines

An inflammation 20-Plex Human ProcartaPlex Panel (EPX200-12185-901, Affymetrix eBioscience, Vienna, Austria) was used to detect the expression of 20 cytokines and chemokines. They were granulocyte colony-stimulating factor (GM-CSF), intercellular cell adhesion molecule-1 (ICAM-1), interferon-*α* (IFN-*α*), IFN-*γ*, interleukin-1*α* (IL-1*α*), IL-1*β*, IL-4, IL-6, IL-8, IL-10, IL-12p70, IL-13, IL-17A, interferon-induced protein-10 (IP-10), monocyte chemoattractant protein-1 (MCP-1), macrophage inflammatory protein 1*α* (MIP-1*α*), MIP-1*β*, sE-selectin, sP-selectin, and tumor necrosis factor-*α* (TNF-*α*).

### 2.8. Statistical Analysis

One-way analysis of variance was used to evaluate the differences among the three groups. Continuous variables were compared using the Wilcoxon rank sum test between the two groups. Fisher's exact test compared categorical variables. Correlations were performed using a nonparametric Spearman test. A two-sided *P* value < 0.05 indicated a significant difference. The threshold logarithmic LDA score for discriminative features was 2. Statistical analyses were conducted using GraphPad Prism 6.0 (La Jolla, CA, USA). Multivariate analysis was performed using SPSS 24.0 (IBM SPSS). Correlations analysis was performed by R i386 software (v.4.0.5).

In addition, an index was introduced to measure the degree of dysbiosis. This index was calculated based on the ratio of the relative abundance of *Enterococcaceae* family to that of *Bifidobacteriaceae* family. The ratio was calculated as follows:

(1) $$\begin{array}{c} \text{Ratio}=\frac{\log_{10}\;\left(100\times Enterococcaceae+1\right)}{\log_{10}\;\left(100\times Bifidobacteriaceae+1\right)}. \end{array}$$

## 3. Results

### 3.1. Patient Characteristics

Altogether, 74 male patients with HCC were included. The average age was 61.00 (53.00–65.00) years. Of them, the early group included 7 patients with stage 0 and 12 with stage A HCC; the intermediate group included 37 patients with stage B HCC; and the terminal group included 8 patients with stage C and 10 with stage D HCC. Among them, 37 patients had HBV and the others had HCV. The demographics and laboratory results are listed in Table 1 and Supplementary Table 1.

### 3.2. Gut Microbial Diversity Decreased with HCC Progression

**Figure 1:**
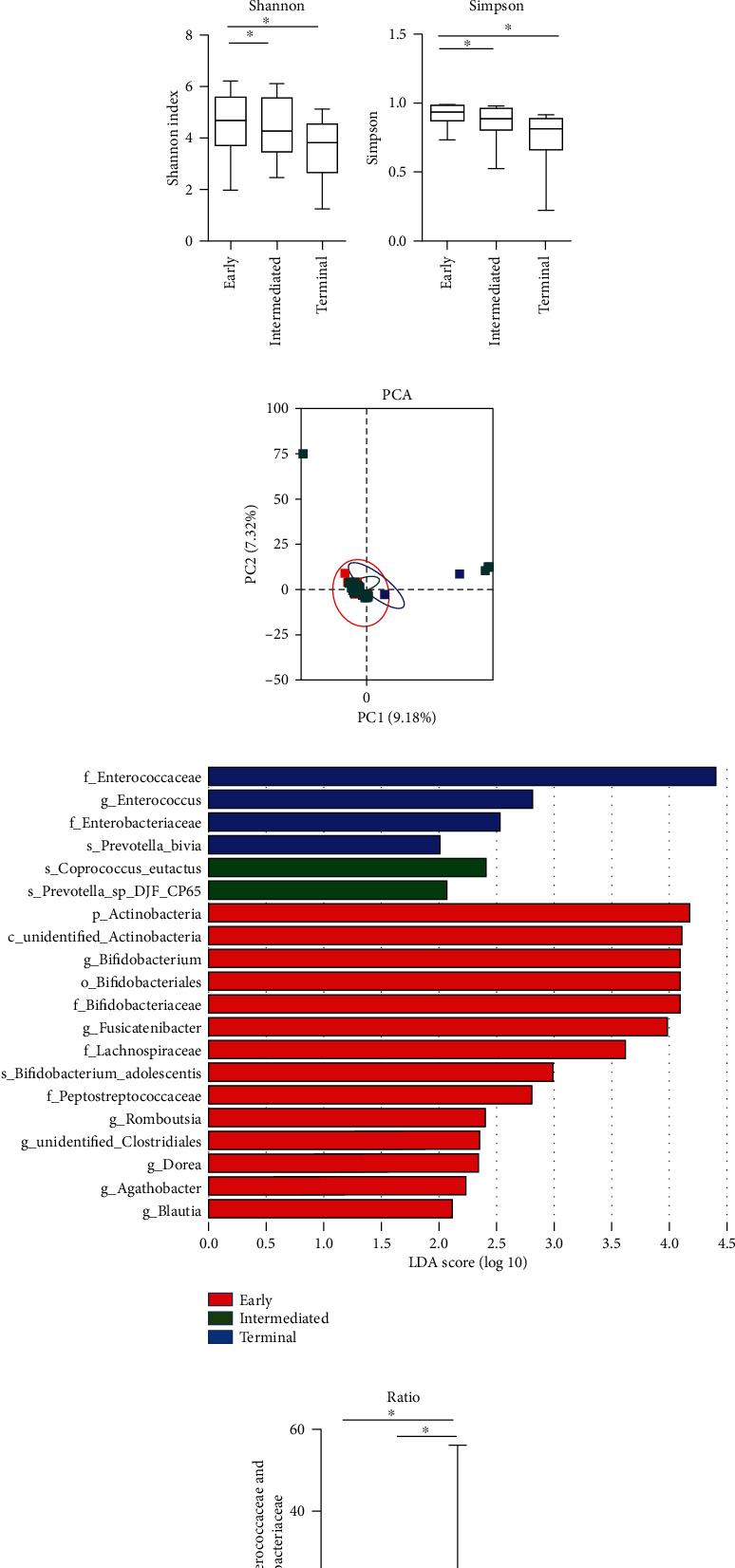
Gut microbiota richness, profiles, and compositions shifted in patients with hepatocellular carcinoma (HCC) progression. Microbial *α*-diversity showed decreased (a) Observed species and (b) Chao1 index in patients with HCC progression, indicating reduced richness. (c) Shannon index and (d) Simpson index decreased significantly with HCC progression. This diminished number of bacteria was accompanied by decreased evenness. The box plots show the smallest and largest values, 25% and 75% quartiles, the median, and outliers. Significant difference (*P* < 0.05) was indicated by an asterisk. (e) Comparison of principal component analysis (PCA) using weighted UniFrac distance showed that the overall fecal microbiota composition (*β*-diversity) distinguished bacterial profiles among early, intermediate, and terminal groups of patients with HCC. Each dot represented one sample, and the distance between the samples represented the difference in community composition of the samples. (f) Histogram of the linear discriminant analysis (LDA) scores for differentially abundant taxa among patients with HCC in the early, intermediate, and terminal groups. Red indicates early group HCC, green indicates intermediated group HCC, and blue indicates terminal group HCC. (g) Change in the ratio of *Enterococcaceae* to *Bifidobacteriaceae* with HCC progression.

The OTU number in early, intermediate, and terminal groups was 322 ± 68, 314 ± 76, and 303 ± 66, respectively. Observed species and Chao1 indexes decreased significantly from early to intermediate and terminal groups (Observed species, *P* = 0.023 and 0.038; Chao1, *P* = 0.013 and 0.042; Figures 1(a) and 1(b)), while evenness reduced (Shannon, *P* = 0.027 and 0.039; Simpson, *P* = 0.017 and 0.028; Figures 1(c) and 1(d)). The results suggested that the alterations of OTUs were evenly contributed with HCC progression.

### 3.3. Gut Microbial Profiles and Compositions Shifted with HCC Progression

**Figure 2:**
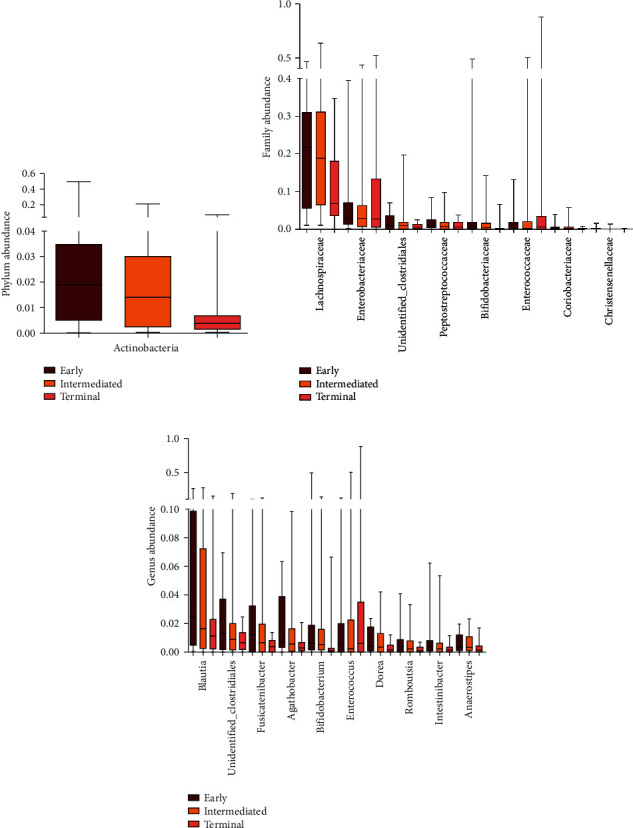
Difference in the abundance of gut microbiota in patients with different groups at the (a) phylum level, (b) family level, and (c) genus level. The difference in the abundance of presented taxon was significant (all *P* values were less than 0.05). The box presented the 95% confidence intervals; the line inside denoted the median, and the symbol “+” denoted the mean value. Red indicates early group HCC, orange indicates intermediated group HCC, and pink indicates terminal group HCC.

The PCA of the *β*-diversity index allowed a separation between any two groups, suggesting that the bacterial profiles of each group were distinguished (Figure 1(e)). The different taxa among the three groups identified by LDA analysis are summarized in Figure 1(f). And the ratio of *Enterococcaceae* to *Bifidobacteriaceae* family significantly increased from the early group to the terminal group (1.5 vs. 9.6, *P* = 0.034; Figure 1(g)). The abundance of *Actinobacteria* phylum (4.46% vs. 0.88%, *P* = 0.020; Figure 2(a)), *Bifidobacteriaceae* family (3.52% vs. 0.56%, *P* = 0.003; Figure 2(b)), and *Bifidobacterium* genus (3.51% vs. 0.56%, *P* = 0.003; Figure 2(c)) significantly decreased in the terminal group compared with the early group. Meanwhile, the abundance of *Enterococcaceae* (1.6% vs. 13.4%, *P* = 0.022), *Enterococcus* genus (1.6% vs. 13.4%, *P* = 0.022), and *Enterobacteriaceae* family (7.7% vs. 10.3%, *P* = 0.046) markedly increased with disease progression. *Lachnospiraceae*, *Peptostreptococcaceae*, unidentified *Clostridiales*, *Coriobacteriaceae*, and *Christensenellaceae* families were enriched in the early group; however, they significantly decreased or were even undetectable in the terminal group (all *P* values < 0.05). Correspondingly, at the genus level, five genera belonging to the *Lachnospiraceae* family (*Blautia*, *Fusicatenibacter*, *Agathobacter*, *Anaerostipes*, and *Dorea*), one genus belonging to the *Clostridiales* family (unidentified *Clostridiales)*, and two genera belonging to the *Peptostreptococcaceae* family (*Romboutsia* and *Intestinibacter*) were also enriched in the early group and decreased or were even undetectable in the terminal group. All the alterations of the short-chain fatty acid- (SCFA-) producing bacteria mentioned earlier contributed to the decrease in richness along with HCC progression.

### 3.4. Gut Damage Increased in Patients in the Terminal Group

**Figure 3:**
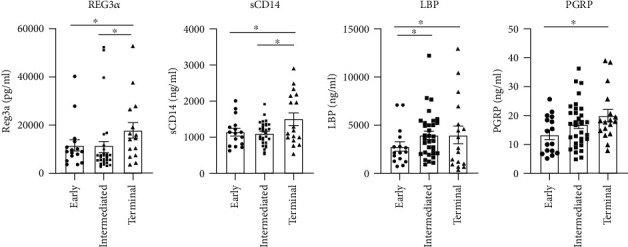
Assessment of gut barrier integrity marker and plasma level of microbial translocation markers in patients with HCC progression. (a) The level of gut damage marker of regenerating islet-derived protein 3*α* (REG3*α*) significantly increased in patients in the terminal group compared with the early and intermediate groups. The levels of Gram-negative bacterial translocation markers, (b) soluble CD14 (sCD14) and (c) lipopolysaccharide-binding protein (LBP), also elevated in patients with HCC in the terminal group. (d) The levels of Gram-positive translocation marker, peptidoglycan recognition proteins (PGRPs), showed that an increased tendency with HCC progression even significantly elevated in patients with HCC in the terminal group compared with the early group. Each dot represented one sample. Significant difference (*P* < 0.05) was indicated by an asterisk.

REG3*α* is a well-known marker of gut damage [21]. The plasma levels of REG3*α* significantly increased in patients in the terminal group (17,830 ± 3257 pg/mL) than in the early (11,591 ± 2388 pg/mL, *P* = 0.025) and intermediate groups (10,881 ± 2298 pg/mL, *P* = 0.023; Figure 3(a)).

### 3.5. Plasma Levels of Microbial Translocation Markers Elevated in Patients in the Terminal Group

Plasma sCD14 elevated in the terminal group (1504 ± 174 ng/mL) compared with the early (1148 ± 100 ng/mL, *P* = 0.048) and intermediate groups (1115 ± 53 ng/mL, *P* = 0.046; Figure 3(b)). The LBP level elevated in the intermediate (3984 ± 412 ng/mL) and terminal groups (4037 ± 911 ng/mL) compared with the early group (2818 ± 488 ng/mL, *P* = 0.035 and 0.025; Figure 3(c)). The PGRP level significantly increased in the terminal group (20.01 ± 2.17 ng/mL) compared with the early group (13.41 ± 1.62 ng/mL, *P* = 0.018; Figure 3(d)).

### 3.6. Cytokine and Chemokine Levels

**Figure 4:**
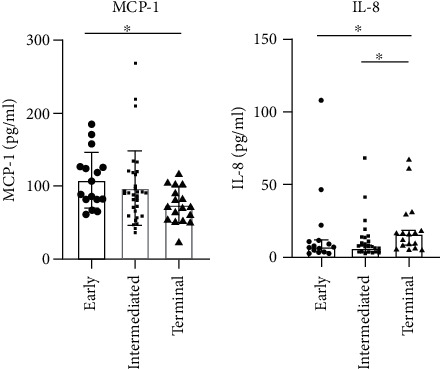
Evaluation of plasma level of inflammatory markers in patients with HCC progression. (a) The plasma level of monocyte chemoattractant protein-1 (MCP-1) showed a decreased tendency with HCC progression, which significantly reduced in the terminal group compared with the early group. (b) The plasma level of IL-8 significantly increased in patients with HCC in the terminal group compared with other groups. Each dot represented one sample. Significant difference (*P* < 0.05) was indicated by an asterisk.

The T cell immune response-related cytokines and chemokines, including IFN-*γ*, IL-4, IL-12p70, IL-13, and IL-17A, showed a decreased tendency from the early to the intermediate and terminal groups, but with no significant differences. The MCP-1 level decreased significantly in the terminal group (74.69 ng/mL) compared with the early (108.26 ng/mL, *P* = 0.005) and intermediate groups (90.70 ng/mL, *P* = 0.015; Figure 4(a)). The levels of chemokines, including GM-CSF, sE-selectin, and sP-selectin, tended to decrease in the terminal group without significant differences. The levels of other proinflammatory cytokines, including IL-1*α*, IL-6, IL-10, IP-10, MIP-1*α*, and MIP-1*β*, increased in the terminal group, but showed no significant difference. The IL-8 level significantly increased in the terminal group (16.31 ng/mL) compared with the early (7.42 ng/mL, *P* = 0.04) and intermediate groups (6.80 ng/mL, *P* = 0.004; Figure 4(b)). These results suggested that HCC progression was characterized by an elevated inflammatory response and suppressed T cell immune responses. The plasma levels of 20 cytokines and chemokines are summarized in Supplementary Table 2.

### 3.7. Relationship between HCC Progression, Gut Damage, Microbial Translocation, and Inflammatory Cytokines in Patients with HCC

**Figure 5:**
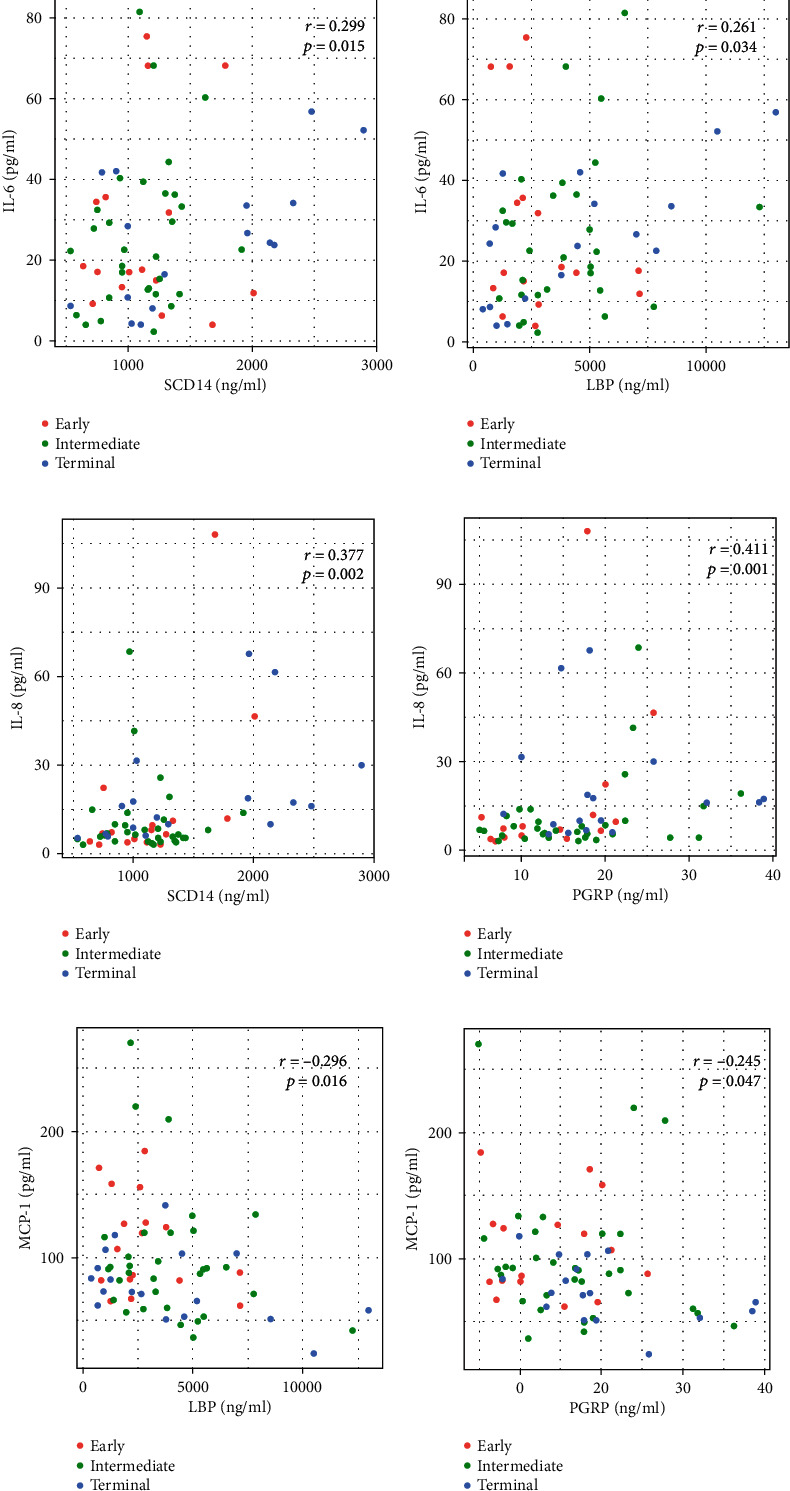
Plasma levels of IL-6, IL-8, and MCP-1 were associated with the markers of microbial translocation. Plasma levels of IL-6 correlated with those of (a) sCD14 and (b) LBP. IL-8 levels correlated with (c) sCD14 and (d) marker of Gram-positive bacterial translocation peptidoglycan recognition proteins (PGRPs). Level of MCP-1 inversely correlated with that of (e) LBP and (f) PGRP. Each dot represented one sample. Red indicates early group HCC, green indicates intermediated group HCC, and blue indicates terminal group HCC.

The IL-6 and IL-8 levels positively correlated with markers of microbial translocation, but did not correlate with REG3*α* or bacterial changes. The IL-6 level positively correlated with the sCD14 level (*r* = 0.299, *P* = 0.015) and the LBP level (*r* = 0.261, *P* = 0.034; Figures 5(a) and 5(b)). They were markers of Gram-negative bacterial translocation. The IL-8 level correlated with the sCD14 (*r* = 0.347, *P* = 0.004) and PGRP (*r* = 0.411, *P* = 0.001; Figures 5(c) and 5(d)) levels, which was a marker of Gram-positive bacterial translocation. The MCP-1 level inversely correlated with the LBP (*r* = −0.296, *P* = 0.016) and PGRP levels (*r* = −0.245, *P* = 0.047; Figures 5(e) and 5(f)). No correlation was found between plasma TNF-*α* level and any of the aforementioned indexes.

### 3.8. The Ratio of *Enterococcaceae* to *Bifidobacteriaceae* Correlated with the Levels of REG3*α* and Markers of Bacterial Translocation

Both *α*-diversity and *β*-diversity could distinguish terminal HCC groups from the other two groups, so did gut damage and microbial translocation markers. Hence, whether gut-associated microbiota changes correlated with the loss of gut barrier integrity and circulating microbial translocation during HCC progression was evaluated. The LDA level revealed a decreased abundance of *Bifidobacteriaceae* family and increased abundance of *Enterococcaceae* family along with HCC progression. The ratio of *Enterococcaceae* to *Bifidobacteriaceae* was associated with the expression of gut damage marker REG3*α* (*r* = 0.366, *P* = 0.003) and bacterial translocation markers, sCD14 (*r* = 0.322, *P* = 0.008), LBP (*r* = 0.386, *P* = 0.001), and PGRP (*r* = 0.405, *P* = 0.001; Table 2). However, the ratio did not correlate with levels of IL-6 and IL-8.

## 4. Discussion

The findings of this study first showed that alterations in gut microbiota, elevated gut damage, and bacterial translocation were associated with HCC progression. Gut dysbiosis of HCC was characterized by increased abundance of *Enterococcaceae* and *Enterobacteriaceae* and decreased abundance of *Bifidobacteriaceae* and SCFA-producing bacteria. Elevation of inflammatory response and suppression of T cell immunity induced by microbial products might be one of the mechanisms of HCC progression.

The data in the present study showed that gut microbial richness decreased significantly in the terminal group compared with the early group. The decrease in richness was mainly due to the reduction in the abundance of SCFA-producing bacteria. The result was consistent with that of a previous study. The study showed that *α*-diversity significantly reduced in patients with BCLC stage C and D HCC compared with patients in stage A HCC [5]. Another similar study showed that the abundance of SCFA-producing family members declined significantly in patients with HCC compared with patients with hepatitis B [4]. SCFA-producing bacteria are known to participate in the process of fermenting diet fibers to SCFAs [22]. SCFAs are a major energy source of the intestinal enterocytes and are essential for maintaining the tight junction and intestinal barrier integrity [23]. However, in this study, the abundance of SCFA-producing bacteria did not correlate with the expression of the serum markers of gut damage and microbial translocation. It was speculated that multiple mechanisms might be involved in SCFA production and did not necessarily depend only on the change in the abundance of SCFA-producing bacteria.

Besides the changes in gut microbiota mentioned earlier, another predominant change was that the abundance of *Bifidobacteriaceae* decreased along with HCC progression accompanied by an increase in the abundance of *Enterococcaceae*. *Bifidobacteria* can reinforce gut barrier function, reduce mucosa inflammation, and protect the liver from injury [24]. Administration of *Bifidobacteria* could mitigate diethylnitrosamine- (DEN-) induced hepatocarcinogenesis in mouse models [25]. *Enterococcus* is regarded as pathogenic bacteria. The overgrowth of *Enterococcus* leads to the release of large amounts of polysaccharide A and LPS, which, in turn, increases gut permeability and facilitates microbial translocation. In this study, the ratio of *Enterococcaceae* to *Bifidobacteriaceae* correlated significantly with the expression of the serum markers of gut damage, microbial translocation, and HCC progression. Therefore, the ratio was considered an ideal quantitative marker of gut dysbiosis in patients with HCC. The ratio of abundance of *Firmicutes* to *Bacteroidetes* [26] or ratio of abundance of genus *Bifidobacterium* to the family *Enterobacteriaceae* was associated with the progression of chronic liver diseases [27]. However, both the ratios were similar among the three groups in this study. More studies should be performed in the future to confirm these results.

The changes in gut damage and microbial translocation biomarkers were subsequently detected due to the close relationship between gut dysbiosis and microbial translocation. REG3*α* is a C-type lectin antimicrobial peptide secreted into the gut lumen by Paneth cells [28]. REG3*α* invades into the bloodstream when gut barrier integrity is damaged [29]. Hence, the circulating level of REG3*α* is well accepted as a marker of gut damage. Previous studies reported that increasing REG3*α* level was always observed in patients with enteropathies, such as Crohn's and celiac diseases, ulcerative colitis, and graft-versus-host disease [29–31]. The results of this study suggested that gut damage worsened gradually along with HCC progression and facilitated the transfer of more microbial products into the bloodstream [32, 33]. This study was the first to show the relationship between gut damage and HCC progression.

This study also found that bacterial translocation increased with HCC progression. LBP is a well-known marker of bacterial translocation [34]. sCD14 is a marker of Gram-negative bacterial translocation, which is secreted by activated myeloid cells after LPS stimulation [35]. PGRP is a peptidoglycan recognition protein and a marker of Gram-positive bacterial translocation [10]. The present study results showed an elevation in the levels of the aforementioned markers with HCC progression. Moreover, increased LBP and sCD14 levels correlated with IL-6 levels, while increased sCD14 and PGRP levels correlated with IL-8 levels. In theory, elevated sCD14 and LBP levels indicated increased circulation of LPS. LPS resulted in the release of proinflammatory cytokines, such as IL-6 and IL-8, by T cells. Chronic inflammation contributed to liver damage and HCC progression [36, 37]. Therefore, it is reasonable that bacterial translocation correlated with HCC progression. However, no relationship was found between gut permeability and microbial translocation. This might be due to bacterial translocation via different mechanisms other than gut integrity. On the contrary, the damage of gut barrier alone might also not be sufficient to cause bacterial translocation, unless additional mechanisms in the complex interactions between host and microbes in the gut failed. A similar result was also observed in patients with alcohol use disorder. In this study, no correlation with intestinal permeability marker was observed, although the levels of all the three markers elevated significantly [10].

Another finding of this study was the elevation of inflammatory response profile during HCC progression, represented by IL-6 and IL-8. IL-8 is a key factor in neutrophil recruitment and activation, which also promoted angiogenesis and metastasis [38, 39]. One previous study found that high serum IL-8 was one of the meaningful predictive cytokines in patients with HBV- and HCV-related HCC [40]. Moreover, as a T cell-attracting chemokine, MCP-1 level decreased significantly in the terminal group, indicating a reduction in T cell immune response. The levels of other cytokines, such as IFN-*γ*, the signature cytokine of the Th1 cell; IL-4 and IL-13, the signature cytokines of the Th2 cell; and IL-17, the signature cytokine of the Th17 cell, reduced simultaneously but without significant differences. These results reflected comprehensive immunity suppression in terminal HCC. The results were in accordance with an *ex vivo* study. The study showed that bacterial extracts from patients with HCC microbiota elicited a T cell-immunosuppressive phenotype [41]. It is well known that SCFAs could promote and regulate the differentiation and apoptosis of Treg cells [42]. According to the results of a previous study and this study, a distinctive microbiome, characterized by decreased abundance of SCFA-producing bacteria, could modulate the peripheral immune response and result in HCC progression.

One limitation of this study was that no causation relationship could be deduced. Whether the alterations of gut microbial profiles, gut permeability, or bacterial translocation was the reason or the result of HCC progression needs further validation in animal models. The other limitation of this study was the lack of geographical representation of the study cohort. In a big hospital in Beijing, most patients came from North China. The third limitation was the lack of age-matched healthy controls. However, this study was designed to evaluate and compare microbiome dysbiosis in different stages of HCC and their relationship with gut damage and microbial translocation. In consideration of the effect of sex on gut microbiota, only male patients were included. The cause of HCC was only HBV and HCV, so the results need further validation in terms of other causes. Finally, the T cell function should be detected directly to confirm the results in this study.

## 5. Conclusions

This study indicated that the dysbiosis of gut microbiota, together with increased gut permeability and microbial translocation, was associated with elevated circulating inflammatory response and reduction in T cell response during HCC progression. The findings obtained in this study also suggested that gut microbiota might be a potential target for HCC treatment and intervention, especially in terminal HCC.

## Figures and Tables

**Table 1:** Clinical characteristics of patients with hepatocellular carcinoma (HCC).

Clinical and pathological indexes	Early group (*n* = 19)	Intermediate group (*n* = 37)	Terminal group (*n* = 18)	p1^#^	p2^#^	p3^#^
Age (years)	61.00 (53.00-65.00)	59.00 (53.00-63.00)	59.00 (47.75-65.75)	1	1	1
ALT (U/L)	30.10 (22.40-39.30)	30.20 (18.85-45.35)	42.15 (27.90-57.28)	1	0.462	0.148
AST (U/L)	31.30 (25.50-45.00)	36.50 (26.65-55.95)	65.45 (52.43-122.50)	1	**<0.001**	**<0.001**
TBIL (*μ*mol/L)	18.50 (15.40-30.10)	25.90 (14.35-42.90)	57.00 (34.55-155.50)	1	**0.001**	**0.005**
DBIL (*μ*mol/L)	9.50 (5.90-14.00)	10.80 (7.70-16.55)	35.45 (19.78-144.20)	0.905	**<0.001**	**0.001**
GGT (U/L)	38.85 (19.00-68.50)	57.30 (38.40-123.80)	164.90 (92.53-240.30)	0.684	**<0.001**	**0.003**
ALP (U/L)	79.75 (60.90-99.53)	97.30 (77.40-115.70)	158.55 (120.80-271.50)	0.276	**<0.001**	**<0.001**
ALB (g/L)	34.40 (30.50-40.30)	34.40 (31.60-38.00)	32.05 (27.18-35.10)	1	0.202	0.152
CRE (*μ*mol/L)	67.60 (52.40-77.60)	61.40 (52.35-66.90)	66.90 (53.93-85.65)	0.669	1	0.223
CHE (U/L)	3866.50 (2975.00-5406.00)	3651.00 (3001.00-4964.00)	1764.50 (1289.00-2892.00)	1	**<0.001**	**<0.001**
TBA (*μ*mol/L)	34.00 (14.23-42.25)	19.30 (6.90-57.10)	53.00 (27.00-133.70)	1	0.097	**0.006**
WBC (10*E*4/L)	3.68 (1.91-4.47)	4.20 (2.69-5.57)	5.06 (3.37-6.39)	0.25	**0.041**	0.25
HGB (g/L)	129.00 (109.00-138.00)	123.00 (105.00-149.00)	105.00 (96.25-132.50)	0.473	0.26	0.398
PLT (10*E*9/L)	87.00 (57.50-97.50)	82.00 (53.5-119.50)	93.50 (47.00-148.50)	1	1	1
INR	1.21 (1.07-1.39)	1.19 (1.09-1.36)	1.20 (1.11-1.32)	1	1	1
MELD score	60.47 (56.36-62.13)	60.12 (56.90-63.55)	64.71 (62.23-67.03)	1	0.307	0.408
Child-Pugh						
A	13 (0.684)	21 (0.568)	1 (0.056)	0.397	**<0.001**	**<0.001**
B	6 (0.316)	16 (0.432)	7 (0.389)	0.397	0.737	0.759
C	0 (0.000)	0 (0.000)	10 (0.556)	—	**<0.001**	**<0.001**

One-way analysis of variance was used to evaluate the difference among the three groups. Continuous variables were compared using Wilcoxon rank sum test between the two groups. Fisher's exact test compared categorical variables. ALT: alanine aminotransferase; AST: aspartate aminotransferase; TBIL: total bilirubin; DBIL: direct bilirubin; GGT: gamma glutamyl transpeptidase; ALP: alkaline phosphatase; ALB: albumin; CRE: creatinine; CHE: cholinesterase; TBA: total serum bile acid; WBC: white blood count; HGB: hematoglobin; PLT: platelets; INR: prothrombin time international standardized ratio; MELD: model for end-stage liver disease. Values indicate median and 25%-75% percentile. ^#^p1 referred to early vs. intermediate, p2 referred to early vs. terminal, and p3 referred to intermediate vs. terminal.

**Table 2:** Correlation between the ratio of *Enterococcaceae* and *Bifidobacteriaceae* or different cytokines and plasma levels of REG3*α* or markers of microbial translocation in HCC patients.

	Ratio of *Enterococcaceae* and *Bifidobacteriaceae*	IL-6	IL-8	MCP-1
REG3*α*	**r** = 0.366 **P** = 0.003	*r* = 0.035 *P* = 0.783	*r* = 0.081 *P* = 0.520	*r* = −0.201 *P* = 0.106
sCD14	**r** = 0.322 **P** = 0.008	**r** = 0.299 **P** = 0.015	**r** = 0.347 **P** = 0.004	*r* = −0.183 *P* = 0.142
LBP	**r** = 0.386 **P** = 0.001	**r** = 0.261 **P** = 0.034	*r* = 0.102 *P* = 0.414	**r** = −0.296 **P** = 0.016
PGRP	**r** = 0.405 **P** = 0.001	*r* = 0.198 *P* = 0.111	**r** = 0.411 **P** = 0.001	**r** = −0.245 **P** = 0.047

MCP-1: monocyte chemoattractant protein-1; REG3*α*: regenerating islet-derived protein 3*α*; sCD14: soluble CD14; LBP: lipopolysaccharide-binding protein; PGRP: peptidoglycan recognition protein.

## Data Availability

The data of clinical characteristics, relative abundance of gut microbiota, and plasma levels of REG3*α*, sCD14, LBP, PGRPs, cytokines, and chemokines of individuals used to support the findings of this study are included within the Supplementary Tables.

## Abbreviations

HBV: Hepatitis B virus
HCC: Hepatocellular carcinoma
HCV: Hepatitis C virus
LBP: Lipopolysaccharide-binding protein
LPS: Lipopolysaccharide
MT: Microbial translocation
OTUs: Operational taxonomy units
PGRPs: Peptidoglycan recognition proteins
REG3*α*: Regenerating islet-derived protein 3*α*
sCD14: Soluble CD14
SCFAs: Short-chain fatty acids.

## Additional Points

*Lay Summary/Key Points*. This study revealed that gut microbiota compositional and functional shifts correlated with hepatocellular carcinoma (HCC) progression, especially in the terminal stage. The mechanism might include elevated gut permeability and microbial translocation relevant to dysbiosis. The accompanying systemic inflammatory increase and immune suppression appeared to promote HCC progression. An increased ratio of *Enterococcaceae* to *Bifidobacteriaceae* could serve as a biomarker of dysbiosis in HCC progression.

## Supplementary Materials

Supplementary 1 Supplementary Table 1: detailed information of study subjects.

Supplementary 2 Supplementary Table 2: concentrations of 20 plasma cytokines and chemokines in the early, intermediated, and terminal groups of HCC patients.
