# Supplementary material for: Dysbiosis of Gut Microbiota Promotes Hepatocellular Carcinoma Progression by Regulating the Immune Response
Source: J Immunol Res. 2021 Oct 20;2021:4973589. doi: 10.1155/2021/4973589 (PMC8551979; doi:10.1155/2021/4973589)
Supplement: Supplementary 1 — Supplementary Table 1: detailed information of study subjects. [file 4973589.f1.pdf]

Supplementary Table 1. Detailed information of study subjects

**Lab number**	**Gender**	**Age**	**Group**	**Etiological classification**	**Record Number**	**Collection data**	**GM-CSF (pg/mL)**	**ICAM-1 (pg/mL)**	**IFN-gamma (pg/mL)**	**IFN-alpha (pg/mL)**	**IL-1alpha (pg/mL)**	**IL-1beta (pg/mL)**	**IL-10 (pg/mL)**	**IL-12p70 (pg/mL)**	**IL-13 (pg/mL)**	**IL-17A (pg/mL)**	**IL-4 (pg/mL)**	**IL-8 (pg/mL)**	**IP-10 (pg/mL)**
HCE1	male	55	early	HCV	298636	20180318	11.8	8E+05	19.55	1.42	3.62	2.32	1.58	22.36	23.65	11.31	24.4	107.77	191.72
HCP2	male	59	terminal	HCV	298866	20180408	19.79	2E+05	12.08	1.26	1.02	2.67	2.54	21.35	17.81	10.09	17.86	8.88	72.33
HCP3	male	70	intermediate	HCV	299738	20180407	73.9	2E+05	27.06	2.98	1.95	7.47	4.68	41.87	50.96	21.2	32.66	6.7	57.43
HCE4	male	62	early	HCV	299484	20180427	59.23	46616	21.88	2.65	1.64	5.12	3.9	34.61	37.86	17.6	24.24	7.26	44.37
HCL8	male	48	intermediate	HBV	300427	20180427	80.04	6E+05	30.7	4.54	1.93	7.09	6.63	48.14	47.38	28.02	28.03	9.73	38.71
HCP10	male	59	intermediate	HCV	300404	20180513	50.88	3E+05	21.88	3.03	1.8	6.37	7.03	38.06	38.3	19.32	30.6	6.51	71.64
HCL14	male	70	terminal	HCV	299668	20180518	<=0	1E+06	17.74	1.03	0.51	2.01	1.77	21.85	30.14	15.09	17.33	5.52	64.3
HCE15	male	56	early	HBV	301034	20180518	14.01	2E+05	15.17	1.42	0.61	1.56	1	22.52	19.34	15.28	13.66	6.89	90.18
HCM19	male	69	terminal	HBV	301130	20180523	<=0	4E+05	5.7	0.04	1.21	1.11	0.91	12.05	35.83	5.86	3.36	6.23	45.43
HCE37	male	71	early	HCV	302108	20180622	38.69	2E+05	17.48	4.09	0.59	5.04	2.06	21.35	40.27	25.84	36.56	11.15	75.2
HCP39	male	68	intermediate	HCV	271886	20180623	44.71	65069	14.39	2.73	2.01	6.62	3.7	37.02	35.14	19.32	21.7	4.45	33.31
HCP40	male	63	intermediate	HCV	297857	20180627	24.79	4E+05	9.27	1.21	1.49	2.47	10.38	19	18.73	11.51	27.05	25.83	173.27
HCP41	male	62	intermediate	HCV	301645	20180627	<=0	8E+05	7.35	0.36	5.76	0.83	2.35	13.03	2.38	7.38	9.16	41.66	126.34
HCP44	male	69	intermediate	HCV	297948	20180628	27.85	99679	10.03	9.15	0.96	3.3	2.49	20.67	22.82	12.72	16.79	4.45	60.17
HCM45	male	59	early	HBV	298989	20180707	9.34	7E+05	9.52	1.99	1	2.55	2.73	14.02	18.73	8.85	21.87	22.31	245.67
HCP49	male	61	intermediate	HCV	300648	20180630	16.06	3E+05	21.62	2.48	1.78	3.7	1.15	27.94	23.92	16.06	24.07	68.41	77.56
HCP50	male	59	intermediate	HCV	301861	20180703	NA	NA	NA	NA	NA	NA	NA	NA	NA	NA	NA	NA	NA
HCM59	male	55	intermediate	HCV	300264	20180713	6.48	5E+05	14.91	1.88	0.71	2.39	1.39	18	19.94	14.3	15.7	10.26	39.91
HCE63	male	34	early	HBV	302770	20180708	<=0	8E+05	9.27	1.03	1.93	1.71	1.96	12.21	14.19	11.11	8.33	46.61	78.94
HCM65	male	60	intermediate	HBV	2E+08	20180708	9.34	3E+05	11.57	1.26	0.55	2.47	1.1	19.33	19.94	11.41	13.09	3.25	35.5
HCE66	male	65	intermediate	HBV	302838	20180713	NA	NA	NA	NA	NA	NA	NA	NA	NA	NA	NA	NA	NA
HCL67	male	75	terminal	HCV	290857	20180714	26.34	6E+05	12.08	1.21	2.79	2.86	6.58	17	22.25	14.01	19.63	67.7	113.95
HCM70	male	53	intermediate	HCV	291578	20180714	50.34	98114	20.97	3.15	1.91	6.04	3.51	38.4	37.63	18.37	28.03	4.29	38.64
HCE71	male	68	early	HCV	289084	20180713	17.97	1E+06	18.78	2.56	0.91	3.9	2.73	29.82	28.14	19.79	21.53	4.08	13.77
HCE76	male	44	early	HBV	276904	20180721	112.9	2E+05	38.79	7.42	3.22	15.9	11.52	82.99	62.55	38.62	43.22	9.69	34.97
HCP120	male	59	terminal	HCV	303822	20180928	70.27	5E+05	42.19	3.25	1.96	6.88	4.29	38.92	36.28	22.14	35.94	17.59	71.7
HCL122	male	59	terminal	HCV	301861	20180922	52.5	5E+05	19.29	2.09	1.47	4.1	3.51	25.73	26.07	15.38	22.72	18.84	43.66
HCM125	male	68	intermediate	HBV	253272	20181110	45.86	5E+05	19.42	2.92	1.11	4.63	3.46	33.93	32.56	19.51	17.68	5.06	36.76
HCM127	male	63	intermediate	HCV	297857	20181024	NA	NA	NA	NA	NA	NA	NA	NA	NA	NA	NA	NA	NA
HCE128	male	66	early	HCV	298535	20181107	37.43	6E+05	21.1	1.79	1.22	3.82	1.58	27.94	27.37	15.48	24.57	7.59	52.47
HCEE129	male	54	early	HBV	304337	20180829	6.48	1E+05	6.97	1.19	0.52	1.67	1.72	16.83	17.18	8.01	9.56	3.34	42.3
HCP130	male	61	early	HBV	228639	20181031	9.34	3E+05	8.88	0.7	0.59	1.71	1.1	11.72	13.84	8.64	7.49	5.17	25.85

HCM130	male	61	intermediate	HBV	228639	2018/6/12	<=0	4E+05	10.67	0.89	1.19	1.86	1.48	13.52	13.49	11.11	10.37	3.52	57.73
HCP131	male	45	terminal	HBV	305430	20181009	24	3E+05	9.27	1.73	2.15	3.34	2.93	23.37	19.04	13.12	19.97	10.05	79.53
HCM132	male	58	intermediate	HCV	298803	20181205	115.8	4E+05	45.59	7.88	3.38	17.4	13.11	81.54	74.47	42.19	49.7	8.71	38.2
HCM133	male	51	intermediate	HBV	221697	20181223	115.1	1E+05	50.84	8.96	4.34	20.2	12.91	104.6	85.02	46.24	52.3	8.31	28.76
HCL137	male	59	terminal	HBV	235895	20181212	87.61	1E+05	29.27	4.38	2.07	8.41	6.83	38.4	47.38	25.01	30.28	6.11	24.66
HCM139	male	51	intermediate	HBV	281741	20181102	26.34	5E+05	26.8	3.56	1.35	4.3	2.59	35.3	28.64	21.95	18.57	7.48	44.19
HCM140	male	63	intermediate	HCV	299484	20181205	39.31	50703	13.62	2.2	1.37	4.02	2.83	26.75	31.12	13.91	19.28	5.92	49.53
HCEE141	male	34	early	HBV	302770	20180902	NA	NA	NA	NA	NA	NA	NA	NA	NA	NA	NA	NA	NA
HCL143	male	53	terminal	HBV	265453	20180119	2.65	8E+05	11.06	0.96	1.04	2.12	2.01	18	14.19	8.22	14.22	30.28	38.56
HCL145	male	68	terminal	HCV	250134	20180829	6.48	2E+05	10.54	0.94	0.62	1.26	1	13.36	13.49	9.89	6.18	16.36	26.57
HCM147	male	61	intermediate	HCV	298645	20181128	30.74	2E+05	16.97	1.64	1.13	2.39	2.06	19.17	22.54	13.91	16.43	15.14	68.16
HCE148	male	66	intermediate	HBV	243943	20181013	60.23	63096	19.55	2.27	1.54	5.16	5.16	32.9	33.98	17.22	24.74	5.71	31.41
HCM149	male	52	intermediate	HBV	305233	20180928	34.83	3E+05	12.85	1.28	1.08	3.74	3.02	26.07	22.25	13.71	11.94	13.8	27.17
HCM149	male	52	intermediate	HBV	305233	2018/10/9	NA	NA	NA	NA	NA	NA	NA	NA	NA	NA	NA	NA	NA
HCE150	male	65	early	HBV	304052	20180928	107.1	2E+05	45.59	9.69	3.51	16.2	13.86	80.46	77.94	38	45.3	12.23	54.4
HCM155	male	52	intermediate	HBV	276959	2018/3/1	<=0	2E+05	6.21	<=0	0.45	1.19	2.25	7.5	7.5	4.3	6.18	4.36	64.13
HCM160	male	51	intermediate	HCV	265809	2018/7/7	44.71	2E+05	28.49	3.69	2.14	7.09	2.83	45.35	43.47	23.72	28.03	8.73	73.62
HCP162	male	42	terminal	HBV	310232	2018/3/22	34.17	2E+05	14.91	1.75	2.25	3.74	4.48	27.26	32.08	14.69	24.07	61.6	83.26
HCE168	male	53	early	HCV	299915	2018/6/16	30.74	1E+05	18.13	2.01	0.83	3.42	2.54	26.41	26.07	17.12	21.01	3.99	76.77
HCM170	male	64	intermediate	HBV	245472	2018/12/23	10.61	4E+05	8.75	0.26	1.05	1.41	1.39	17	9.68	9.06	9.56	11.51	71.04
HCM170	male	64	intermediate	HBV	245472	2018/4/13	2.65	3E+05	4.94	<=0	0.63	0.97	0.53	11.55	0.23	5.75	6.62	6.89	45.22
HCP170	male	64	terminal	HBV	245472	2018/9/23	9.34	4E+05	6.21	0.18	1.05	1.59	1.15	14.02	8.84	6.95	7.49	12.47	45.47
HCP172	male	44	terminal	HBV	307902	2018/1/6	16.06	7E+05	13.11	1.57	1.63	2.24	2.06	16	22.25	13.22	15.7	31.75	109.69
HCM173	male	37	intermediate	HCV	278285	2018/5/10	17.03	46981	10.67	0.61	0.66	1.86	1.67	13.69	16.53	9.06	13.85	5.66	66.6
HCP181	male	47	terminal	HBV	308250	2018/5/9	37.43	2E+05	22.39	2.48	1.66	4.75	3.41	29.82	29.15	16.93	27.7	17.74	42.44
HCM182	male	55	intermediate	HBV	258618	2018/3/25	41.15	2E+05	20.33	2.94	1.35	5.54	3.02	32.21	31.12	18.18	25.9	4.06	30.52
HCM187	male	54	intermediate	HBV	242349	2018/4/20	2.65	2E+05	9.78	0.68	0.59	1.71	1.2	15.84	17.81	3.14	11.16	5.57	55.46
HCEE198	male	61	early	HCV	301281	2018/1/6	10.61	54712	7.86	1.03	0.67	2.98	1.77	18.67	14.88	12.32	8.54	3.18	28.84
HCM199	male	54	intermediate	HCV	311202	2018/5/4	37.43	2E+05	33.57	4.86	0.63	6.5	1.87	42.22	39.4	25.19	40.22	3.13	45.29
HCM200	male	55	intermediate	HCV	302388	2018/4/14	95.64	64331	27.19	3.67	1.94	7.73	7.67	38.4	51.35	22.7	33.45	5.87	30.36
HCM202	male	57	intermediate	HBV	311206	2018/5/3	24.79	3E+05	16.58	0.85	1.45	2.32	2.83	20.34	24.47	11.51	14.22	19.51	80.61
HCM205	male	60	intermediate	HBV	241432	2018/9/6	37.43	1E+05	18.78	2.56	1.23	4.06	2.06	28.79	27.88	16.45	21.53	14.07	62.22
HCL206	male	62	terminal	HCV	256724	2018/11/14	<=0	3E+05	8.75	0.7	0.51	1.41	0.62	16.67	13.84	9.47	13.66	7.15	60.1
HCL207	male	63	terminal	HCV	301645	2018/5/9	NA	NA	NA	NA	NA	NA	NA	NA	NA	NA	NA	NA	NA
HCM214	male	59	intermediate	HBV	305589	2018/10/13	17.97	1E+06	16.2	1.9	0.47	1.37	2.54	19.33	19.04	14.11	16.06	6.91	60.07
HCM216	male	53	intermediate	HBV	281181	2018/1/6	86.78	2E+05	33.96	5.78	2.81	10.6	9.09	62.58	58.92	31.45	40.52	8.38	52.68
HCEE217	male	51	early	HCV	166652	2018/9/20	90.86	3E+05	45.99	7.85	3.64	15.2	12.47	74.35	68.88	44.17	45.6	8.33	33.88
HCP218	male	65	terminal	HBV	303974	2018/3/6	44.71	1E+05	8.75	1.48	1.5	3.38	2.93	20.84	19.34	12.52	16.79	10.17	57.06

HCE219	male	72	early	HCV	302108	2018/6/22	NA	NA	NA	NA	NA	NA	NA	NA	NA	NA	NA	NA	NA
HCEE228	male	61	early	HCV	299246	2018/11/22	NA	NA	NA	NA	NA	NA	NA	NA	NA	NA	NA	NA	NA
HCL229	male	48	terminal	HBV	311218	2018/5/2	31.44	4E+05	23.17	3.11	1.68	4.38	3.22	34.61	32.08	19.89	23.06	16.31	30.36
HCEE231	male	63	early	HCV	310126	2018/3/29	17.97	1E+05	10.8	1.3	0.79	3.02	1.96	22.69	21.11	11.51	17.15	4.36	74.83

MCP-1 (pg/mL)	IL-6 (pg/mL)	MIP-1alpha (pg/mL)	MIP-1beta (pg/mL)	sE-Selectin (pg/mL)	sP-Selectin (pg/mL)	TNF-alpha (pg/mL)	REG3a (pg/mL)	sCD14 (ng/mL)	PGRPs (ng/mL)	LBP (ng/mL)	Alanine aminotransferase (ALT)	Aspartate aminotransferase (AST)	Total bilirubin(TBIL)	Direct bilirubin (DBIL)	Gamma glutamyl transferase (GGT)	Alkaline phosphatase (ALP)	Cholinesterase (CHE)	Total serum bile acid (TBA)	Na+	White Blood Cell (WBC)
119.1	3.95	14.38	42.83	22078	2E+05	60.98	10899	1681	17.9	2707	39.3	45	17.6	9.5	76.6	80	3604	33.1	138	3.84
72.43	10.7	6.72	27.76	16343	2E+05	36.05	6844	997.7	13.76	2252	82.2	112.1	29.6	14.9	85.4	146	3171	22.6	137	6.15
97.04	36.13	22.22	25.48	17464	7E+05	61.97	5433	1382	14.15	3422	19.7	26.7	23.4	10.8	45.5	103	4083	15.9	137	3.76
126.3	34.56	8.84	24.4	18126	7E+05	46.96	5284	741.7	14.59	1861	38	24.2	30.1	10.5	63.2	84.4	3931	5.7	136	4.47
100.2	40.24	30.43	31.89	11593	2E+06	84.21	11575	930.9	12.06	2076	31.7	34.2	9	4.8	217	126	6754	3	143	5.8
92.28	32.45	11.13	25.84	18968	6E+05	60.49	51001	757	16.72	1273	73.5	57.7	13.9	6.4	57.2	41.4	4964	5.8	140	5.26
61.66	8.6	50.3	11.58	36168	5E+05	70.36	14079	532.4	13.3	710.6	20.5	42.4	154.9	141.3	143.3	93.2	1330	11	128	12.3
65.61	6.38	9.88	31.75	18346	1E+05	60.32	10190	1274	19.57	1252	43.1	32.8	27.3	14	13.8	97.4	3571	42.7	141	1.88
106.4	3.95	7.48	30.36	16249	19807	22.88	14533	1100	20.82	1046	22	53.2	61.6	52.3	60.6	94.9	1294	118	141	3.38
184.8	31.92	9.04	22.44	15262	45985	34.32	14441	1333	5.229	2805	15.7	31.5	47.3	34.9	19	60.9	1943	34.9	137	1.98
81.58	29.22	7.48	30.57	17386	4E+05	31.89	6420	852.4	17.64	1680	16.2	44	16.1	8.8	38.6	77.4	4142	45.5	140	4.2
119.8	11.38	6.04	39.17	21232	5E+05	48.31	4078	1222	22.32	2808	35.3	36.5	26.4	15.9	69.4	121	4093	6.1	145	4.16
72.54	<=0	6.66	78.68	34470	2E+06	47.63	5428	1012	23.32	3328	80	122.2	43.7	23.7	59	88.6	2156	86.6	139	5.45
209.9	20.75	4.35	13.68	11749	80530	26.62	7377	1222	27.73	3917	16.5	26.3	14.6	8.2	24.4	92.2	3534	9.4	144	1.44
158	17.15	6.21	25.91	32960	79632	37.77	9781	757	20.04	1305	27.7	25.5	15.4	7	242.3	65.4	5524	30.8	140	3.68
219.4	22.5	16.21	54.45	23978	3E+05	57	5213	975.8	23.96	2425	49.8	104	18.1	8.7	22.1	81.3	4702	33.7	137	3.23
NA	NA	NA	NA	NA	NA	NA	NA	NA	NA	NA	497.2	96.1	75.4	54.7	118.9	116	2653	104	136	7.23
90.6	10.7	8.48	18.72	20539	2E+05	57.67	7190	847.7	22.36	1141	18	54.6	90.5	74.3	162.7	141	1659	131	138	2.29
88.41	11.71	9.04	64.36	29825	4E+05	22.52	27910	2009	25.7	7141	22.4	22	13.1	6.9	16.6	58.6	7672	7.1	142	4.49
91.29	6.38	3.06	14.16	17059	37996	37.08	5615	586.5	7.32	5643	23.2	26.6	25.9	8.4	22	92	1573	19.1	136	4.55
NA	NA	NA	NA	NA	NA	NA	NA	NA	NA	NA	25.1	36.5	29.9	15.6	63.7	97.3	3651	57.1	142	2.68
103.4	26.47	16.34	93.31	15733	2E+05	36.05	37559	1966	18.23	6994	137.2	852.4	157.4	152.9	205.9	208	1259	175	120	5.57
59.65	39.22	8.94	24.4	15313	3E+05	45.95	10925	1119	31.23	3866	12.8	18.5	31.5	15.9	47.1	71	3478	8.3	143	2.21
61.55	17.45	13.94	35.66	26705	3E+05	60.98	8426	1113	15.42	7138	670.3	1083	23.9	14	53.8	119	5367	18.1	135	4.11
107	67.92	16.5	34.84	18024	5E+05	71.67	3692	1157	21.26	1564	47.6	59.4	8.4	5.1	207.9	64.4	####	45.9	146	4.84
65.72	34.04	15.03	36.81	16775	7E+05	75.11	17775	2335	38.93	5177	33.5	63	17.1	8.4	126.4	126	1703	51	138	2.41
50.98	33.51	10.56	26.41	14057	3E+05	64.94	26603	1954	17.92	8523	59.3	84.3	239.5	156.9	157.2	146	2790	190	135	5.49
86.9	22.21	9.78	20	10985	3E+05	60.65	9861	537.8	7.717	5318	10.5	21.3	43	9.8	22	53	4422	26.6	140	2.3
NA	NA	NA	NA	NA	NA	NA	NA	NA	NA	NA	32.1	28.1	17.9	9.7	57.3	102	4307	6.2	146	4.19
82.2	35.61	10.85	18.34	12936	65538	50.66	9091	815.7	7.848	2152	35.8	59.5	34.5	18	16.1	102	936	42.1	141	1.44
67.31	14.96	9.39	55.4	25944	8E+05	30.67	4423	1227	7.114	2179	36.1	29.4	18.3	8.5	65.8	99.3	4254	40.5	141	3.82
81.89	17.15	7.75	18.19	12482	41302	29.79	40157	1007	10.04	4430	29.1	31.3	23.3	10.9	42.3	78.1	2585	76.1	133	1.31

53.27	12.71	10.46	19.25	12274	29970	28.39	52081	1162	18.96	5476	35.5	41.4	31.5	8.6	22	102	1573	19.1	133	2.67
50.98	16.53	10.08	35.05	13658	7E+05	43.91	10130	1292	19.46	3799	38.1	50.1	42.9	21.4	94.9	119	2880	34.7	142	3.34
119.4	67.92	18.59	38.5	11810	1E+06	442.7	3250	1207	20.06	3976	11.1	17.6	14.5	5	15.9	95.8	7962	2	143	4.15
92.67	81.27	21.07	36.34	11986	3E+05	75.92	16217	1094	9.055	6523	36.2	51.4	42.8	13.6	136.7	158	3104	37.4	139	4.83
82.51	41.76	12.06	21.7	10461	7E+05	64.61	3755	791.7	15.6	1269	48.9	149.5	52.4	36.4	275.2	249	1473	71.5	130	7.09
121.2	17.15	15.41	34.77	15785	2E+06	84.21	3205	948.1	11.93	5038	22.9	17.5	11.7	5.5	48.3	80.5	7158	12.3	141	7.05
132.9	27.85	6.77	19.62	18377	3E+05	32.94	4622	726.4	12.88	4984	36.2	36.9	19.9	8.4	50.5	72.8	3529	27.1	136	3.2
NA	NA	NA	NA	NA	NA	NA	NA	NA	NA	NA	30.1	24.8	18.5	3.7	38	80	2456	38.8	142	4.29
23.76	52.07	9.29	54.58	21943	8E+05	45.61	16414	2900	25.76	10491	56.6	138.1	37.3	27.1	572.9	434	2359	31.1	140	5.25
53.27	42.01	5.4	17.2	14386	41047	39.83	10388	908.8	32.09	4618	28.9	67.3	68.2	51.2	159.1	191	1203	55	134	2.91
56.75	3.95	9.69	31.96	16872	2E+05	54.84	7869	655.6	31.73	1999	30.2	31.3	7.9	5.1	65.7	80.9	4065	7.1	139	6.12
42.39	33.25	5.57	21.33	11854	1E+06	50.99	39805	1433	17.86	12276	9.3	15	9.8	6	30.5	51.5	3443	19.9	143	12
134.1	22.5	5.4	22.29	17394	53920	40.17	16762	1914	9.8	7864	63.2	59.1	18.3	11.1	181.1	137	5336	17.3	143	6.72
NA	NA	NA	NA	NA	NA	NA	NA	NA	NA	NA	28.8	34.8	11.1	7.2	153.5	139	5954	2.5	139	5.46
170.7	68.15	18.43	42.03	8500	6E+05	93.91	9067	1785	18.54	771	26	28.7	15.7	5.9	37	59.3	8229	6.9	140	7.2
70.89	8.6	8.48	16.81	3147	17523	19.62	7454	1349	13.32	7748	25	22.4	13.6	6.1	46.7	79.3	5028	26.7	140	4.6
79.91	38.71	11.04	26.41	23627	9E+05	66.59	5663	1460	20.78	3618	34.7	71.3	29	15.6	150.8	113	2281	92.9	136	4.21
103.5	23.65	8.89	44.28	21704	3E+06	59.33	7489	2181	14.81	4499	92.1	120.9	280.3	205.2	562.8	339	3295	135	136	7.14
81.79	13.36	10.27	27.2	20800	1E+06	52.67	3687	956.8	6.304	863.9	121.6	154.1	17.4	8	39.3	117	3105	50.3	137	2.81
93.45	15.28	4.66	26.13	14527	2E+05	42.56	5925	1254	8.258	2124	131.9	113.8	40.2	24.5	85.6	114	1842	68.4	133	2.7
115.7	<=0	1.97	11.66	16116	17763	28.21	4222	1015	5.564	989.5	40.7	46.2	14.2	8.2	123.8	113	1894	19.3	139	1.09
83.03	7.88	3.75	12.39	12152	51499	26.27	15949	1195	7.897	399.5	20.5	36.7	14.2	10.7	178.5	171	2061	27.5	134	1.84
117.8	4.38	17.33	42.43	17878	42964	51.33	4221	1030	9.989	1478	41.9	54.8	470.6	343.5	170.7	231	4834	167	136	3.62
59.41	2.13	3.48	38.16	20337	6E+05	41.87	3728	1212	12.6	2780	40.9	45.2	70.6	51.9	63.6	115	3468	207	136	1.47
72.86	28.4	16.75	32.44	14086	8E+05	67.74	52797	997.8	18.51	980.7	35.4	36.1	16.7	7.7	228.7	133	2928	13.7	137	4.86
66.29	29.49	9.09	20.74	14083	5E+05	55.51	7630	1356	10.39	1429	23.3	23.5	50.1	17.2	38.4	62.8	8097	18.8	140	4.97
88	11.38	8.38	30.15	14075	1E+05	37.94	8072	1411	20.93	2073	9.4	23	56.5	24.2	32.4	78.6	2677	4.2	134	4.53
127.3	9.31	<=0	13.2	11253	46824	21.26	8038	718.1	6.773	2852	29.2	37.7	34.2	14.3	38.4	79.5	3298	29.8	138	1.49
83.74	13.04	9.88	63.61	16692	5E+05	74.94	7578	1169	16.81	3212	22.6	32.6	33.8	13.3	30.2	110	3252	97.7	136	1.22
48.9	44.26	9.78	20.3	9658	5E+05	62.14	2651	1329	17.88	5259	16.5	25	12.5	4.1	21.2	57.3	8468	5.6	143	3.63
46.78	36.39	18.22	28.96	19940	9E+05	55.84	NA	1297	36.18	4470	22.4	35.9	49.5	39.2	143.3	169	1344	20.2	133	6.14
36.27	18.36	11.5	33.06	8255	1E+06	63.3	NA	949.8	11.07	5047	74.3	48.8	39.4	14	50.5	54.2	3409	6.9	142	3.69
71	<=0	7.85	58.33	23280	37111	37.08	18956	769.1	17.74	2676	42.4	63.3	64.4	34.5	36	82.2	1356	105	142	4.23
NA	NA	NA	NA	NA	NA	NA	NA	NA	NA	NA	24.9	63.6	43.4	27.9	55.1	83.8	954	133	132	5.36
269.5	4.79	11.32	25.91	32054	6E+06	79.34	3532	777.7	4.947	2190	60.3	131.1	188.2	135.1	758.6	451	4713	71.8	139	5.68
90.8	60.34	14.12	43.09	12206	2E+05	75.11	6928	1625	17.23	5512	60.1	57.3	20.2	10.8	114.4	111	3001	18.6	138	6.05
86.29	75.33	16.83	33.13	12743	96946	68.73	7205	1147	10.19	2287	11.3	21.4	27.1	12.3	373.8	52.2	3802	16.6	136	7.66
91.88	24.22	2.98	22.07	7490	2E+05	26.98	NA	2139	16.93	710.7	48.6	127.2	36.2	21.9	173.1	464	1826	25.5	138	4.28

NA	NA	NA	NA	NA	NA	NA	NA	NA	NA	NA	15.7	31.5	47.3	34.9	19	60.9	1943	34.9	137	1.98
NA	NA	NA	NA	NA	NA	NA	NA	NA	NA	NA	19.8	27.8	12.4	5	22.5	100	3947	36.9	1337	1.91
58.45	56.83	13.68	28.4	17430	2E+06	76.9	27792	2480	38.42	13006	45.4	108.8	72.6	48.5	485.3	466	1275	40.7	128	10.2
124.1	18.36	5.28	19.7	23973	4E+05	31.02	13163	643.2	7.964	3775	38.6	30.5	9.5	4.1	38	84.8	4152	4.5	142	3.13

Hematoglobin (HGB)	Platelets (PLT)	Prothrombin Time (PT)	Prothrombin activity (PTA)	Prothrombin time international standardized ratio (INR)	Creatinine (CRE)	Child grading of liver function	Model for end-stage liver disease (MELD)	Firmicutes phylum	Bacteroidetes phylum	Actinobacteria phylum	Fusobacteria phylum	Tenericutes phylum	Enterococcaceae family	Lachnospiraceae family	Bifidobacteriaceae family	Enterobacteriaceae family	Peptostreptococcaceae family	unidentified_Clostridiales family	ratio of Enterococcaceae and Bifidobacteriaceae
134	96	12.1	89	1.08	52.4	B	56.165	0.435	0.528	0.029	2E-04	1E-03	0.005	0.11	0.019	0.003	0.0469	0.0355	0.385844
115	101	14.7	65	1.3	70	B	62.998	0.414	0.489	0.072	0.001	2E-04	0.025	0.171	0.066	0.013	0.012	0.0044	0.613885
123	79	11.6	96	1.03	44.2	A	55.083	0.745	0.146	0.069	1E-04	3E-05	0.03	0.198	0.058	0.027	0.0285	0.0086	0.726126
128	94	11.6	94	1.03	51.5	A	57.507	0.301	0.481	0.042	4E-04	0.004	0.002	0.167	0.035	0.167	0.0047	0.0049	0.098449
155	233	11.1	101	233	61.5	A	115.34	0.827	0.103	0.014	0.001	2E-04	7E-04	0.636	0.007	0.052	0.003	0.0045	0.133727
153	119	11.3	98	1.01	64.4	A	56.498	0.833	0.094	0.073	0	6E-05	2E-04	0.421	0.066	2E-04	0.0085	0.017	0.007822
76	44	17.3	52	1.52	479.3	C	89.506	0.951	0.014	0.006	2E-04	0	0.662	0.051	0.004	0.019	0.0059	0.0025	11.43166
129	50	14.1	70	1.25	78.5	B	63.351	0.942	0.013	0.024	1E-04	1E-04	0.131	0.259	0.019	0.013	0.016	0.0042	2.488594
99	39	17.3	53	1.52	62. 2	C	66.399	0.902	0.046	0.026	0.001	3E-04	0.036	0.189	0.014	0.01	0.0184	0.0252	1.74618
109	50	15.6	63	1.39	75.8	B	66.293	0.781	0.118	0.027	0	3E-04	0.002	0.355	0.011	0.052	0.0682	0.0708	0.184629
139	70	12.1	89	1.08	66	A	58.042	0.83	0.049	0.029	3E-04	0	8E-04	0.22	0.016	0.078	0.0074	0.0019	0.079938
164	110	12.7	82	1.13	63.5	A	60.058	0.592	0.269	0.054	0	3E-04	0.018	0.233	0.039	0.057	0.0163	0.0149	0.65467
140	57	13.4	76	1.19	54.2	B	61.032	0.523	0.056	0.024	1E-04	2E-04	0.028	0.208	0.016	0.059	0.0164	0.0305	1.383806
102	82	11.7	93	1.04	62.9	A	56.786	0.405	0.159	0.031	2E-04	8E-04	0.01	0.197	0.017	0.389	0.0371	0.0153	0.681848
117	88	11.9	91	1.06	74.3	A	58.801	0.495	0.406	0.019	0	6E-05	4E-04	0.271	0.012	0.013	0.0205	0.0571	0.043324
133	68	12.7	83	1.13	66.7	A	59.095	0.751	0.109	0.077	8E-04	2E-04	0.004	0.405	0.017	0.057	0.0354	0.0162	0.312766
130	55	15	66	1.34	67.1	B	66.484	0.852	0.064	0.028	5E-04	0	0.003	0.513	0.02	0.048	0.0157	0.0038	0.258038
115	57	17.2	54	1.53	61.7	B	67.857	0.677	0.089	0.208	6E-04	0.001	0.004	0.337	0.141	0.016	0.0584	0.0321	0.119725
159	123	13.6	75	1.21	67.6	A	58.762	0.199	0.015	0.013	0.644	3E-04	0.02	0.056	0.007	0.055	0.0081	0.0072	2.113139
79	149	13.4	76	1.19	68.8	B	61.334	0.884	0.007	0.024	0.011	4E-04	0.503	0.07	0.014	0.054	0.0109	0.0198	4.500879
142	24	14.9	67	1.33	50.3	A	60.119	0.53	0.006	0.008	0.011	3E-04	0.076	0.053	0.003	0.434	0.0069	0.0092	7.555966
88	201	14	72	1.25	118.7	C	73.978	0.931	0.002	0.009	0.008	3E-04	0.749	0.058	0.004	0.045	0.0205	0.0135	12.44338
109	67	11.6	95	1.04	71	A	60.871	0.706	0.025	0.114	0.016	0.002	0.028	0.16	0.053	0.029	0.0769	0.1954	0.718565
110	65	13.4	77	1.19	80.7	A	62.56	0.822	0.021	0.061	0.015	0.014	0.043	0.393	0.016	0.043	0.062	0.0421	1.753045
160	156	10.3	115	0.92	86.4	A	56.36	0.782	0.104	0.035	4E-04	6E-04	0.023	0.228	0.022	0.071	0.0059	0.0074	1.016017
103	53	11.8	92	1.05	95.7	A	61.523	0.988	0.004	0.002	3E-05	3E-05	0.881	0.038	8E-04	0.003	0.0051	0.0093	56.15333
102	86	11.3	99	1.01	54.3	C	65.677	0.602	0.389	0.006	6E-05	7E-04	0.01	0.348	0.002	9E-04	0.0374	0.0149	3.909689
122	65	14.7	67	1.31	83.9	B	66.241	0.5	0.259	0.023	9E-04	0.004	0.011	0.188	0.011	0.212	0.027	0.0159	1.021036
156	112	12.1	89	1.08	56	A	56.868	0.868	0.087	0.032	0.001	0.006	0.004	0.436	0.015	0.003	0.0175	0.0118	0.347699
106	40	14	69	1.27	57	B	61.346	0.565	0.21	0.005	0.002	0.002	0.016	0.302	0.002	0.199	0.0186	0.0684	5.302609
135	97	14.2	70	1.26	80.2	A	62.126	0.823	0.041	0.052	0.002	0.063	0.003	0.429	0.003	0.013	0.025	0.0295	0.761774
74	17	16.4	59	1.46	55.8	B	61.212	0.866	0.1	0.011	0.002	3E-04	0.032	0.144	0.004	0.018	0.0184	0.0374	3.956727

84	18	15.9	57	1.42	57	B	62.251	0.272	0.62	0.002	3E-05	0	3E-04	0.023	0.002	0.066	0.0008	0.0006	3.956727
125	35	14.3	70	1.28	77.4	B	65.199	0.84	0.113	0.003	0.003	3E-04	0.008	0.194	2E-04	0.036	0.0062	0.0083	30.3405
151	153	12.4	86	1.11	67.4	A	58.153	0.753	0.232	0.012	0	0	1E-04	0.255	0.008	0.003	0.0103	0.0167	0.016768
129	112	12.2	88	1.09	74.1	A	62.972	0.842	0.062	0.062	0.002	0.005	0.007	0.356	0.001	0.023	0.0149	0.1198	4.801387
120	107	12.9	81	1.15	97.7	C	66.995	0.682	0.307	0.005	2E-04	2E-04	0.009	0.323	4E-04	0.002	0.0188	0.0244	18.97477
154	144	11.4	97	1.02	66.4	A	56.247	0.857	0.086	0.02	0.007	0.005	0.027	0.287	0.002	0.019	0.0182	0.0237	6.199251
99	77	12.7	83	1.13	47.5	A	56.197	0.877	0.101	0.01	8E-04	5E-04	0.012	0.447	0.002	0.009	0.0982	0.0831	3.857202
170	139	13.6	75	1.21	72.5	A	60.745	0.372	0.037	0.006	0.283	0.003	0.008	0.219	0.001	0.297	0.0092	0.0139	4.18156
134	183	12.2	88	1.09	55.9	C	59.744	0.459	0.337	0.007	0.03	0.003	0.005	0.176	0.002	0.126	0.0212	0.0128	2.01709
88	44	12.9	81	1.15	84.4	C	66.592	0.82	0.043	0.007	0.035	0.001	0.035	0.084	0.003	0.087	0.0178	0.0139	6.567404
140	148	11.5	96	1.03	48	A	51.749	0.767	0.213	0.009	6E-05	4E-04	0.217	0.135	0.003	0.006	0.0278	0.0248	11.65829
83	230	18.8	48	1.67	131.6	B	67.662	0.768	0.207	0.005	6E-04	0.001	0.035	0.273	0.002	0.008	0.0048	0.0063	10.80537
114	250	13.9	72	1.24	56.2	A	58.533	0.962	0.02	0.006	3E-05	3E-04	0.043	0.144	0.001	0.006	0.0194	0.0117	14.92953
123	420	12.3	86	1.1	49.6	A	54.092	0.106	0.863	0.002	1E-04	0	4E-04	0.012	0.002	0.02	0.0012	0.0008	0.207023
164	120	12	90	1.07	77.6	A	59.397	0.933	0.026	0.009	9E-04	0.002	0.008	0.467	0.003	0.018	0.0114	0.0231	2.123455
153	51	12.6	83	1.12	60.6	A	56.989	0.166	0.785	0.002	0.001	0	2E-04	0.035	0.002	0.006	0.0024	0.0538	0.082844
150	99	13.3	76	1.19	41.6	B	56.934	0.115	0.798	0.001	3E-04	0	2E-04	0.068	0.001	0.076	0.002	0.0026	0.128762
137	233	12.5	84	1.12	52.7	B	67.146	0.561	0.153	4E-04	0.032	0	6E-05	0.046	6E-05	0.095	0.0003	0.0014	1
131	69	15.7	60	1.4	38.6	A	56.094	0.095	0.881	0.001	6E-05	0	2E-04	0.042	1E-03	0.013	0.0008	0.0019	0.201075
63	45	15.9	60	1.41	51.4	B	62.105	0.212	0.66	0.012	0.002	3E-05	4E-04	0.096	0.008	0.092	0.0032	0.0208	0.07153
41	35	13.4	77	1.2	62.3	B	58.191	0.44	0.481	0.014	0.005	0	4E-04	0.128	0.005	0.021	0.0038	0.0022	0.086341
67	57	12.5	82	1.12	68.7	B	58.357	0.106	0.866	1E-03	0.002	0	0	0.033	3E-04	0.007	0.0006	0.0055	0
135	137	14	72	1.25	65.1	B	72.373	0.167	0.751	0.003	7E-04	6E-05	1E-04	0.069	0.001	0.019	0.0005	0.0021	0.092511
112	43	17.6	49	1.58	31.2	B	60.728	0.695	0.23	0.017	5E-04	0.002	0.003	0.216	0.016	0.048	0.0016	0.0037	0.250847
132	119	12.4	84	1.11	47	B	55.229	0.075	0.417	0.002	4E-04	0.002	3E-05	0.014	0.001	0.486	0.0003	0.0004	0.028552
166	110	12.2	88	1.09	78.5	A	64.124	0.047	0.866	0.001	0.003	0	3E-04	0.028	0.001	0.08	0.0004	0.0008	0.244757
108	45	15.3	60	1.37	71.3	B	66.218	0.304	0.319	0.011	6E-05	0	5E-04	0.092	0.004	0.061	0.0014	0.0005	0.129768
107	82	16	59	1.42	49.5	A	61.209	0.182	0.771	9E-04	0.004	0	1E-03	0.039	4E-04	0.009	0.0005	0.0011	2.249041
117	25	15.3	60	1.37	53.3	B	61.473	0.115	0.864	5E-04	6E-05	0	2E-04	0.061	5E-04	0.001	0.0175	0.0076	0.317899
148	120	12.2	88	1.09	57.9	A	55.927	0.154	0.818	0.001	4E-04	1E-04	3E-05	0.115	7E-04	0.003	0.0023	0.0012	0.046962
79	96	14.8	65	1.32	47.3	B	61.359	0.082	0.587	0.015	0.278	2E-04	1E-03	0.026	0.013	0.037	0.0037	0.001	0.113426
119	52	17.3	50	1.54	58.2	B	64.21	0.463	0.325	0.002	7E-04	2E-04	4E-04	0.376	0.001	0.162	0.0002	0.0013	0.309163
106	48	15.5	62	1.38	45.4	C	62.464	0.103	0.649	1E-04	0.006	0	6E-05	0.066	3E-05	0.231	0.0004	0.0035	1.996814
100	58	16.2	55	1.45	60.2	C	64.227	0.088	0.704	0.006	4E-04	0	3E-05	0.022	3E-04	0.155	0.0012	0.0003	0.126392
3.81	108	133.7	75	1.22	61.4	B	68.057	0.243	0.74	0.001	7E-04	3E-04	0	0.027	1E-04	0.01	6E-05	0.0002	0
139	93	13.3	77	1.19	53.7	A	58.011	0.111	0.857	0.002	9E-04	1E-04	6E-05	0.031	0.002	0.003	0.001	0.0011	0.040723
138	98	14.7	68	1.31	55.2	A	60.468	0.367	0.526	0.019	0.022	0	6E-04	0.31	0.019	0.052	0.0055	0.0015	0.05566
104	118	11.9	91	1.06	86.3	B	63.486	0.414	0.046	0.003	0.001	0	1E-04	0.125	3E-04	0.525	0.0274	0.0171	0.403789

109	50	15.6	63	1.39	75.8	B	66.293	0.473	0.028	0.493	0.001	0	0.02	0.011	0.492	0.002	0.0844	0.0221	0.277076
84	78	12.4	86	1.11	44.5	A	53.573	0.314	0.645	5E-04	5E-04	0	2E-04	0.206	2E-04	0.029	0.0017	0.0009	0.834649
136	232	14.6	66	1.3	52.8	C	63.7	0.107	0.873	4E-04	0	0	0.002	1E-03	6E-05	1E-04	0.0018	6E-05	25.83731
132	87	11.1	101	0.99	65	A	54.916	0.109	0.47	3E-04	0.001	0	6E-05	0.016	1E-04	0.396	0.0004	0.0001	0.50159
