# Supplementary material for: Dysbiosis of Gut Microbiota Promotes Hepatocellular Carcinoma Progression by Regulating the Immune Response
Source: J Immunol Res. 2021 Oct 20;2021:4973589. doi: 10.1155/2021/4973589 (PMC8551979; doi:10.1155/2021/4973589)
Supplement: Supplementary 2 — Supplementary Table 2: concentrations of 20 plasma cytokines and chemokines in the early, intermediated, and terminal groups of HCC patients. [file 4973589.f2.docx]

**Supplementary Table 2. Concentrations (pg/ml) of 20 plasma cytokines and chemokines in early, intermediated and terminal group of HCC patients**

| Cytokine | early group  (n=17) | intermediated group (n=32) | terminal group  (n=16) |
| --- | --- | --- | --- |
| GM-CSF | 17.97(9.66,54.10)* | 38.34(11.97,50.75) | 27.22(4.57,41.07) |
| ICAM-1 | 237532.55  (132407.09,656582.49)* | 288938.51  (116497.21,399384.31) | 412679.59  (229802.65,546841.58) |
| IFN-γ | 17.81(9.33,21.69)* | 19.16(10.67,25.57) | 15.68(9.01,20.84) |
| IFN-α | 1.89(1.22,3.73)* | 2.38(0.97,3.46)* | 1.66(0.95,2.29) |
| IL-1α | 0.96(0.63,2.90)* | 1.35(0.77,1.93)* | 1.48(1.03,2.02) |
| IL-1β | 3.22(1.86,5.10)* | 3.88(1.98,6.47)* | 3.31(1.80,4.24) |
| IL-10 | 2.01(1.62,3.61)* | 2.71(1.72,5.04)* | 2.96(1.46,3.90) |
| IL-12p70 | 22.61(17.29,33.41)* | 27.35(18.25,38.31)* | 22.90(16.34,28.54) |
| IL-13 | 24.86(17.57,39.67)* | 30.16(18.81,38.13) | 24.71(16.00,32.08) |
| IL-17A | 15.38(11.16,24.33)* | 15.18(11.44,20.78)* | 13.68(9.68,16.16) |
| IL-4 | 23.31(10.59,33.56) | 22.10(13.28,22.03) | 18.59(13.94,23.57) |
| **IL-8** | **7.42(4.15,11.96)*** | **6.80(4.60,11.20)*** | **16.31(8.02,24.56)*** |
| IP-10 | 53.44(34.15,78.40)* | 51.11(37.12,65.98)* | 59.36(40.50,75.93) |
| **MCP-1** | **108.26(81.82,127.04)** | **90.70(61.31,118.50)*** | **74.69(55.86,97.65)** |
| IL-6 | 17.30(12.12,35.35)* | 24.43(10.87,35.41) | 24.41(8.24,37.90) |
| MIP-1α | 10.41(8.02,14.27) | 9.39(6.20,13.47)* | 10.08(7.10,15.69)* |
| MIP-1β | 31.84(20.39,40.44) | 29.99(20.08,35.95) | 35.01(21.89,43.36) |
| sE-Selectin | 19372.22  (12791.44,25451.13) | 16193.06  (11886.75,18820.45) | 16248.88  (13857.41,19790.95)* |
| sP-Selectin | 333162.75  (69061.50,604202.79) | 335875.88  (167481.29,866652.59)* | 274478.23  (47231.81,748675.30)* |
| TNF-α | 48.39(30.76,60.98) | 55.18(38.50,63.01)* | 49.70(36.05,66.34) |

* Values indicate median and 25%-75% percentile.
